# Flexible Electrocorticography Electrode Array for Epileptiform Electrical Activity Recording under Glutamate and GABA Modulation on the Primary Somatosensory Cortex of Rats

**DOI:** 10.3390/mi11080732

**Published:** 2020-07-29

**Authors:** Xinrong Li, Yilin Song, Guihua Xiao, Jingyu Xie, Yuchuan Dai, Yu Xing, Enhui He, Yun Wang, Shengwei Xu, Lulu Zhang, Duli Yu, Tiger H. Tao, Xinxia Cai

**Affiliations:** 1 State Key Laboratory of Transducer Technology, Aerospace Information Research Institute, Chinese Academy of Sciences, Beijing 100190, China; lxr8118@126.com (X.L.); ylsong@mail.ie.ac.cn (Y.S.); xiaoguihua11@126.com (G.X.); xiejingyu16@mails.ucas.ac.cn (J.X.); hongri1991@126.com (Y.D.); xingyu17@mails.ucas.ac.cn (Y.X.); heenhui_iecas@163.com (E.H.); wangyun_iecas@163.com (Y.W.); swxu@mail.ie.ac.cn (S.X.); 2 University of Chinese Academy of Sciences, Beijing 100049, China; 3 College of Information Science and Technology, Beijing University of Chemical Technology, Beijing 100029, China; llzhang@mail.buct.edu.cn (L.Z.); dyu@mail.buct.edu.cn (D.Y.); 4 State Key Laboratory of Transducer Technology, Shanghai Institute of Microsystem and Information Technology, Chinese Academy of Sciences, Shanghai 200050, China; tiger@mail.sim.ac.cn

**Keywords:** ECoG electrode array, flexible, epilepsy, electrical activity, primary somatosensory cortex

## Abstract

Epilepsy is a common neurological disorder. There is still a lack of methods to accurately detect cortical activity and locate lesions. In this work, a flexible electrocorticography (ECoG) electrode array based on polydimethylsiloxane (PDMS)-parylene was fabricated to detect epileptiform activity under glutamate (Glu) and gamma-aminobutyric acid (GABA) modulation on primary somatosensory cortex of rats. The electrode with a thickness of 20 μm has good flexibility to establish reliable contact with the cortex. Fourteen recording sites with a diameter of 60 μm are modified by electroplating platinum black nanoparticles, which effectively improve the performance with lower impedance, obtaining a sensitive sensing interface. The electrode enables real-time capturing changes in neural activity under drug modulation. Under Glu modulation, neuronal populations showed abnormal excitability, manifested as hypsarrhythmia rhythm and continuous or periodic spike wave epileptiform activity, with power increasing significantly. Under GABA modulation, the excitement was inhibited, with amplitude and power reduced to normal. The flexible ECoG electrode array could monitor cortical activity, providing us with an effective tool for further studying epilepsy and locating lesions.

## 1. Introduction

Epilepsy is a common neurological disorder, affecting about 50 million people worldwide [1,2]. When the excitatory and inhibitory activities are out of balance, the abnormal discharges of neurons cause paroxysmal brain dysfunction, manifested as limb twitching or abnormal conscious behavior [3]. Glutamate (Glu) is a major excitatory neurotransmitter in the central nervous system, which has been related to the onset and propagation of seizures [4,5]. Excessive Glu can cause disturbance of neuronal functional activity and induce seizures [6].

Clinically, electroencephalography (EEG) is recognized as the main basis for diagnosis of epilepsy [7]. It monitors abnormal changes in brain electrical activity through scalp electrodes, which play an important role in detecting and predicting seizures [8]. However, the EEG electrodes have low sensitivity and large artifacts due to interference from the scalp, skull, and dura mater. Electrocorticography (ECoG) is a method to record electrical activity directly from the surface of the cerebral cortex with minimal invasiveness to brain tissues [9,10]. A comparison with EEG shows that ECoG has advantages in spatial resolution, signal amplitude, and fidelity [11]. Thus, ECoG electrodes are becoming increasingly popular for epilepsy study [12].

Commercial ECoG electrodes (Ad-Tech Medical Instrument Corporation, USA), with millimeter electrode diameters (typically 2–3 mm) and pitches, are available to monitor cortical activity and locate the seizure focus during surgical diagnosis [13,14]. In the last decade, in order to obtain higher spatial resolution of cortical signals, there has been an evolution of ECoG into micro-electrocorticography (µECoG) by scaling down the dimensions of ECoG electrodes to micron size [15].

Polydimethylsiloxane (PDMS) [16,17], parylene [18,19], and polyimide (PI) [20,21] have been widely used as substrate materials to fabricate flexible electrodes, reducing tissue damage, and improving recording reliability [22]. These materials are biocompatible and compatible with MEMS fabrication processes. PDMS, with a Young’s modulus of 0.4–1.0 MPa, its mechanical property is more similar to the brain tissue than parylene (4–4.5 GPa) and PI (2.3–2.8 GPa) [23,24]. However, its thermal expansion coefficient differs from metals by an order of magnitude. Microcracks easily occur during sputtering and patterning metals processes [25]. In addition, Adrega’s team found that square apertures of 200 to 30 μm could be patterned in the PDMS insulation layer to expose the underlying metals. The patterning resolution was limited to >20 μm features [26]. The size of electrode recording sites is critical to the spatial resolution and detection performance. Resolution >20 μm greatly limits the possibility of detecting single neuron. While parylene is an excellent solution to the problems faced by PDMS, parylene, with a thermal expansion coefficient of 3.5 × 10^−5^ K^−1^, is relatively more comparable to metals (1.42 × 10^−5^ K^−1^) [25] than PDMS (2.0 × 10^−4^ K^−1^) [27] and oxygen plasma treatment improves the adhesion between parylene and metals [28]. Thus, parylene enables stronger metal adhesion than PDMS. Moreover, parylene is easy to form a 1–2 μm thin layer and create openings by oxygen plasma etching. It is easier to obtain smaller size (even single neuron size) of openings to expose the underlying metals in fabrication processes. Since PDMS and parylene have their own advantages, combining the two is a good choice [29].

Considering the needs for the detection of neural activity, ECoG electrodes should be flexible enough to accommodate the curved structure of the brain, with PDMS as the preferred material. The insulation thickness should be as thin as possible to allow the exposed electrode sites to attach to the nerve cells; 1–2 µm parylene can meet the demand. Therefore, we combined PDMS and parylene. PDMS was selected as the substrate layer. Parylene was selected as the transition layer to enhance adhesion between PDMS and the metal layer, and as an insulation layer to easily create openings.

In this paper, we fabricated a flexible ECoG electrode array based on PDMS and parylene with a high spatial resolution for recording the neural activity on the primary somatosensory cortex of rats and facilitate the detection and localization of cortical lesions.

## 2. Materials and Methods

### 2.1. Reagents and Apparatus

Phosphate-buffered saline (PBS, 0.1 M, pH 7.4) was obtained from Sigma (St. Louis, MO, USA). Saline (0.9% NaCl) was purchased from Shuanghe Corporation (Beijing, China). Gamma-aminobutyric acid (GABA) was purchased from Tocris Bioscience (Bristol, UK). Glu was purchased from Beijing Xinjingke Corporation (Beijing, China).

The modification of flexible ECoG electrode array was carried out on an electrochemical workstation (Gamry Reference 600, Gamry Instruments, Warminster, PA, USA). An animal isoflurane anesthesia machine (RWD520, RWD life science, Shenzhen, China) was used to anesthetize rats during the surgery. The electrical signals were recorded by a homemade neural data recording instrument.

### 2.2. Electrocorticography (ECoG) Electrode Array Fabrication

**Figure 1:**
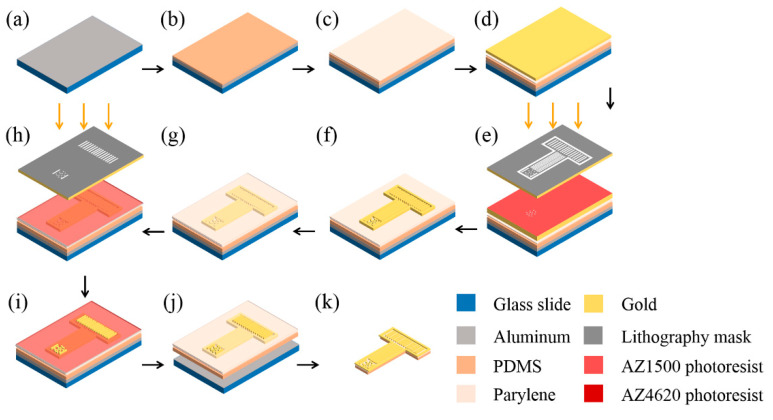
The fabrication processes of the flexible electrocorticography (ECoG) electrode array. (**a**,**b**) Steps to prepare glass slide and polydimethylsiloxane (PDMS); (**c**–**f**) Steps to pattern metal layer; (**g**) Deposit insulation layer; (**h**–**j**) Steps to create openings in insulation layer; (**k**) Release from the glass slide.

The whole fabrication processes were completed in the clean room. The processes are shown in Figure 1 and described as follows:

#### 2.2.1. Preparing Glass Slide and PDMS

A clean glass slide was coated with 1000 Å Al to form a sacrificial layer (Figure 1a). PDMS was mixed with a curing agent at a 10:1 weight ratio, which was used as the substrate material. A 20 μm flexible substrate layer was formed by spin coating the de-aired PDMS on the sacrificial layer at a speed of 1000 rpm (Figure 1b). Then, it was cured on a hot plate for 4 h at 80 °C.

#### 2.2.2. Patterning Metal Layer

To enhance the adhesion between the metal layer and PDMS substrate, a 1.5 μm thin parylene layer was evaporated on PDMS (Figure 1c). Then, the sample was sputtered 300 Å Cr and 2000 Å Au (Figure 1d). After the sample was treated in oxygen plasma, positive photoresist AZ1500 was spun on the metal layer at 1000 rpm for 60 s (Figure 1e). Then, it was cured in an oven at 60 °C. The temperature was strictly controlled at 60 °C, which was the most suitable temperature to form a crack-free photoresist film. Due to the large differences in thermal expansion and contraction rates between PDMS and photoresists, cracks would form when the temperature was high or changed quickly. After lithography, the photoresist was developed in 0.6% NaOH, and hard-baked, at 60 °C. The sites, lines, and pads of the electrode array were protected by the photoresist mask. The exposed metal layer was corroded, leaving the desired electrode features (Figure 1f). Then, the sample was immersed in acetone to remove the photoresist mask. Following treatment in oxygen plasma, the residual photoresist was fully removed.

#### 2.2.3. Depositing Insulation Layer 

The sample was put into a parylene coating machine and 4.5 g parylene-C was evaporated for 6 h to form a 1.5 μm insulation layer (Figure 1g).

#### 2.2.4. Creating Openings in Insulation Layer 

Positive photoresist AZ4620 was spin-coated at 1500 rpm for 60 s and cured in an oven at 60 °C to form a 10 μm thick layer. A lithography mask was used to expose the electrode sites and pads (Figure 1h). After lithography, the sample was developed in 0.6% NaOH, and post-baked at 60 °C. The electrode sites and pads were exposed, while the electrode lines were protected by the photoresist (Figure 1i). The parylene insulation layer above sites and pads was etched by oxygen plasma with a power of 100 W to create openings (Figure 1j). Oxygen plasma could etch both parylene and positive photoresist. It was necessary to ensure that the photoresist mask was thick enough to protect the parylene above electrode lines from etching.

#### 2.2.5. Releasing from the Glass Slide

The sample was immersed in 20% NaOH for 20 min to etch the Al sacrificial layer, and gently released from the glass slide (Figure 1k). Then, electrodes were soaked in deionized water to remove residual NaOH and any other chemicals.

### 2.3. Modification of the ECoG Electrode Array

With the advantage of specific surface area, platinum black nanoparticles (PtNPs) were electroplated onto the electrode sites to decrease the electrode impedance [30]. Nano-modified technology enabled sensitive sensing interface, allowing electrodes to closely contact with cortical cells. PtNPs were electroplated by a three-electrode system including working electrode, Ag/AgCl reference electrode, and Pt counter electrode, using the amperometry method at −1.1 V for 50 s. The electroplating solution was mixed by 24 mM H_2_PtCl_6_ and 2.1 mM Pb(CH_3_COO)_2_. During the electroplating process, ultrasonic vibration was required to uniformly grow the nanoparticles.

### 2.4. Experimental Procedures

**Figure 2:**
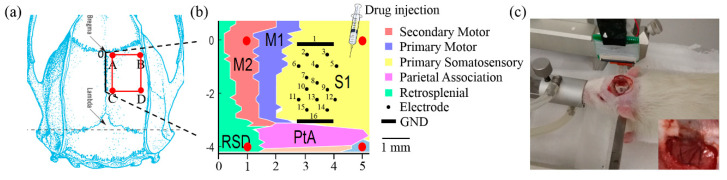
The placement of the ECoG electrode array for neural activities recording. (**a**) Cortical craniotomy location was performed in the red frame; (**b**) Location of electrode sites attached to subdural primary somatosensory cortex of rats. The drug was injected on the primary somatosensory cortex, adjacent to the ECoG electrode array; (**c**) The ECoG electrode array was attached to the cortex of anesthetized rats.

Male Sprague-Dawley (SD) rats weighing 250 g were used in the experiments. All animal experiments were performed with the permission of Beijing Association on Laboratory Animal Care. Two rats were used for these experiments. One rat was used for the Glu and GABA modulation experiment. Another rat was used for the repetitive experiments of Glu-induced seizures. During the surgery, the rat was anesthetized by animal isoflurane anesthesia machine and fixed in a stereotaxic apparatus. A craniotomy of 4 × 4 mm (coordinates relative to bregma, 4 mm posterior, 4 mm lateral) was performed on the skull of the right hemisphere with a dental drill (Figure 2a). The skull and dura mater were removed. The skull nail was placed on the left brain of the skull for grounding. After surgery, the rat was put into a shield cage to shield the external electromagnetic interference. The flexible ECoG electrode array was placed onto the surface of the exposed subdural cortex (Figure 2c). Fourteen microelectrode sites were distributed on the primary somatosensory cortex (Figure 2b), which greatly improved the spatial resolution as compared with the conventional EEG electrodes. In vivo detection of rats from normal to drug-induced epileptiform activity was performed. The drug injection area was adjacent to the array (Figure 2b). The average detection time was over 4 h. The electrical signals were recorded at a sampling frequency of 1 kHz using a homemade neural data recording instrument.

The normal activity was recorded for 30 min to ensure stable recording and exclude interference. Then, Glu (10 μL, 1 mM) was injected in the primary somatosensory cortex of rats to induce neuronal epileptiform activity. As an excitatory neurotransmitter, Glu is involved in triggering and propagating seizures [31]. The accumulation of extracellular Glu can induce epileptiform activity. After recording the excitatory fluctuation for 7 min, Glu (10 μL, 1 mM) was injected the second time. With further absorption and accumulation of Glu, epileptiform activity became intense and lasted about 30 min. During the period of epileptiform activity, GABA (10 μL, 1 mM) was injected into the primary somatosensory cortex. As an inhibitory neurotransmitter, it had an obvious inhibitory effect on neurons [32,33].

## 3. Results

### 3.1. The Characterization of Electrode

**Figure 3:**
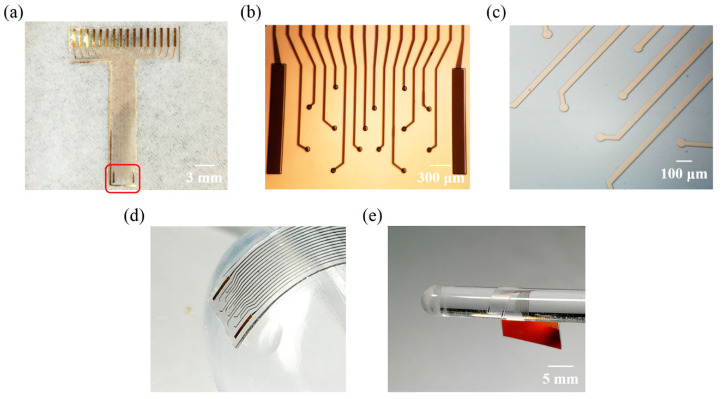
Optical image and the mechanical flexibility of the ECoG electrode array. (**a**) Layout of the electrode; (**b**) Microphotograph of the electrode array; (**c**) Partial enlarged image with clear lines and no cracks; (**d**) The mechanical flexibility was investigated by placing it on a hemisphere ~1.3 cm in diameter; (**e**) Rolling around a cylindrical tube 5 mm in diameter.

The fabrication results of flexible ECoG electrode array are shown in Figure 3. The electrode sites area (3.6 × 2.28 mm) could cover most regions of the primary somatosensory cortex (Figure 3a). It could be used to either monitor lesions or locate the cortical functional area. The interconnecting electrode pads (0.35 × 3 mm) were designed to fit FPC connector (16 channel, 1 mm pitch). The connector required a certain thickness and stiffness to keep electrical connection. Thus, the back of the electrode pad area was stuck to a PI thin slice (0.3 mm total thickness) before being inserted into the connector. The overall thickness of this electrode was about 20 μm, considering that the electrode should be easy to handle during the experiment, to avoid the electrode curling itself due to excessive thinness. The electrode thickness can be varied according to the experimental needs by adjusting the thickness of the underlying PDMS layer in the fabrication process. The electrode consisted of 14 recording sites (60 μm diameter each) and two ground (GND) sites (Figure 3b). The distance between electrode sites (center to center) was 300 and 600 μm, which effectively avoided mutual interference. A partially enlarged image showed that the surface of the electrode was clean, and the lines were smooth with no cracks (Figure 3c). The mechanical flexibility of the ECoG electrode was investigated by placing it on a hemisphere ~1.3 cm in diameter (Figure 3d) and rolling it around a cylindrical tube 5 mm in diameter (Figure 3e). The electrode was well accommodated to the curved structure, which indicated good flexibility. Additionally, for in vivo experiments, the electrode kept a certain bending over 4 h (Figure 2c) and maintained a reliable record. This also reflected the good mechanical flexibility of our electrode.

In this work, the ECoG electrode array satisfied the need for detecting electrical activity of neuronal populations in primary somatosensory cortex.

### 3.2. Impedance Test of the ECoG Electrode Array

**Figure 4:**
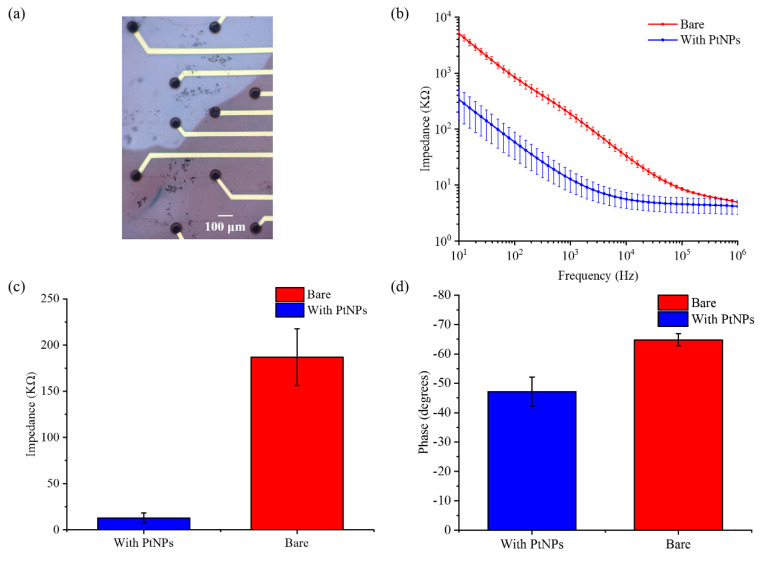
The flexible ECoG electrode array was modified with platinum black nanoparticles (PtNPs). (**a**) Photomicrograph of the electrode array with PtNPs modification; (**b**) Electrode impedance plots before and after PtNPs modification; (**c**) At 1 kHz frequency, the impedance decreased from 186.96 ± 30.62 kΩ to 12.78 ± 5.35 kΩ; (**d**) The phase increased from −64.79 ± 2.01° to −47.17 ± 4.96°.

The surface morphology of the electrode after electroplating PtNPs is shown in Figure 4a. Electrode impedance was a primary concern in detection of weak neural signals. To evaluate the properties of the flexible ECoG electrode array, it was characterized by electrochemical impedance. The impedance of 14 electrode sites (60 μm in diameter) was tested in PBS at room temperature using a three-electrode system. The electrochemical impedance spectroscopy (EIS) was recorded from 10 to 1 MHz using a Gamry electrochemical workstation. Figure 4b clearly shows the average impedance of 14 electrode sites recorded before and after modification. As shown in Figure 4c,d, PtNPs enhanced electrode performance in terms of impedance and phase. The average impedance at 1 kHz was decreased from 186.96 ± 30.62 kΩ to 12.78 ± 5.35 kΩ. The phase angle at 1 kHz was shifted from −64.79 ± 2.01° to −47.17 ± 4.96°. The PtNPs reduced the electrode impedance and simultaneously improved the signal delay, which was beneficial to recording electrical signals.

### 3.3. Characteristics of Electrical Activity under Glutamate (Glu) and Gamma-Aminobutyric Acid (GABA) Modulation

Local field potential (LFP) is a key signal in the study of neuronal communication of the brain [34]. It is a measure of electrical activity in a volume of neuronal tissue [35]. Therefore, it is critical to accurately record LFP to understand the mechanisms of interaction between neuronal populations.

**Figure 5:**
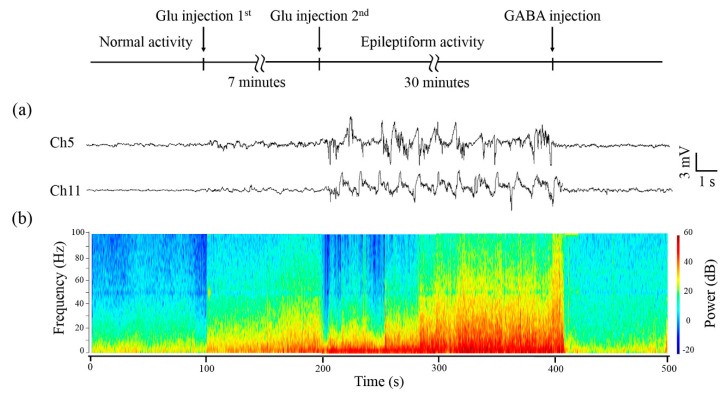
In vivo neural activity recording under modulation of glutamate (Glu) and gamma-aminobutyric acid (GABA). The arrow indicated the time of Glu injection (1st), (2nd), and GABA injection. (**a**) Real-time recording of local field potential (LFP) waveforms; (**b**) Time-frequency power spectrogram analysis.

Glu and GABA were applied to modulate the neural activity of cortical neuronal populations. The flexible ECoG electrode array was applied to capture changes in neural activity of the rat’s primary somatosensory cortex. Figure 5a shows representative channels of real-time LFP (a low pass filter of 200 Hz) waveforms recorded by the ECoG electrode array. The waveforms are divided into different stages, including normal activity, first time Glu injection, epileptiform activity, and GABA inhibitory activity. For normal activity, the amplitude of waveforms fluctuates within 1 mV. After the first Glu injection, the neuronal populations are induced to be excited, with amplitude fluctuating in 2 mV. With the second Glu injection, the amplitude rapidly increases to 6 mV and continues to fluctuate sharply, manifested as hypsarrhythmia rhythm and continuous epileptiform activity. The neuronal populations are more excited, which is related to the accumulation and diffusion of Glu. Hyperexcitability is one of the characteristics of the seizure focus. With GABA injection, the electrical activity tends to be normal. The amplitude falls back to 1 mV, which indicates that GABA has an obvious inhibitory effect on neurons.

Figure 5b displays the time-frequency power spectrogram corresponding to Figure 5a. Power spectrum is a method to analyze LFPs in a frequency domain. It transforms the electrical signal to a spectrum whose power changes with frequency. The power of LFPs in a normal stage is concentrated in 0–5 Hz. With accumulation and spread of extracellular Glu, which destroys the balance of Glu absorption and release, inducing abnormal epileptiform activity, a rapid increase in power at 0–30 Hz is observed. After GABA injection, the power in each frequency band reduces significantly due to the inhibitory effect on neurons [36,37].

These results show that our flexible ECoG electrode array is a promising tool for stable recording of cortical electrical activity. The characteristics of electrical activity are helpful for studying epilepsy and locating lesions.

### 3.4. Frequency Domain Characteristics Analysis of Electrical Signals

**Figure 6:**
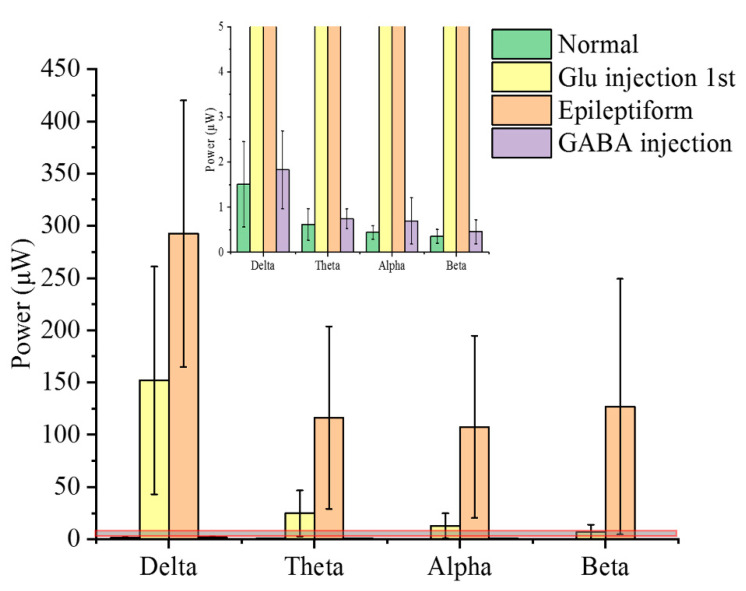
Power analysis in the frequency domain. The power of LFPs (μW) in multiple frequency bands (delta (1–4 Hz), theta (4–8 Hz), alpha (8–13 Hz), beta (13–30 Hz)) at different stages. The error bars represent standard deviation in power from three channels. An enlarged view of the gray area framed by the red line is shown at the top of the figure.

The electrical signals were decomposed into multiple frequency bands (delta (1–4 Hz), theta (4–8 Hz), alpha (8–13 Hz), and beta (13–30 Hz)). Each frequency band was characterized with different amplitudes and rhythms. LFPs’ power in different stages was further analyzed in a frequency domain, as shown in Figure 6 (Figure S1). Power was calculated from each frequency band to get specific features for seizure detection or prediction. The frequency bands with large changes in power were highly correlated with seizures as compared with other frequency bands [38].

The power of Glu-induced excitatory activity is higher than normal activity. Further accumulation of Glu in extracellular fluid induces intense epileptiform activity and excitement transmission, with another significant increase in power. The change in delta band is the most prominent among all frequency bands (repetitive experiment Figure S2). Taking the delta band power as an example, the power is 1.50 ± 0.94 μW for normal activity and increases to 152.03 ± 109.03 μW for the first Glu-induced excitatory activity. Then, it reaches a maximum of 292.47 ± 127.6 μW during the epileptiform activity. Cortical lesions can exhibit abnormal delta activity.

When Glu was injected for the first time, the neural populations showed a slight excitement, which was obviously reflected in the delta band, whereas other frequency bands showed only a small increase. The small increase was not easy to distinguish from the normal state. It was not until the epileptiform state that these frequency bands had obvious changes. The delta band had the highest power, therefore, it could be detected changes early. Seizures are likely to be predicted by detecting changes in delta frequency band. Frequency band characteristics have an inspiring value for the study of epilepsy.

### 3.5. Repetitive Experiment of Glu-Induced Epileptiform Activity

**Figure 7:**
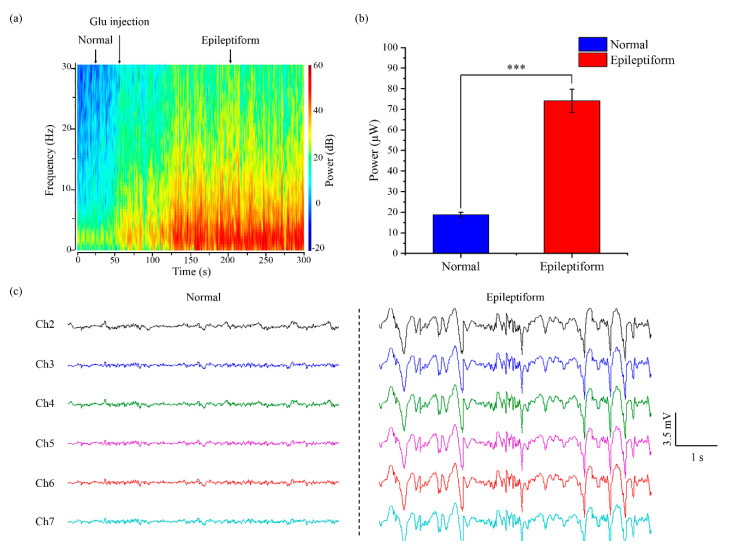
Epileptiform electrical activity recording using ECoG electrode array. (**a**) Time-frequency power spectrum; (**b**) Power analysis of normal and epileptiform activity. (*** *P* < 0.001). The data from six channels were calculated; (**c**) Simultaneous multisite recording with ECoG electrode array.

A repetitive experiment was performed on rats for Glu-induced epileptiform activity. Figure 7a shows the time-frequency power spectrum of Glu-induced seizures. It can be seen from the figure that the activity of neuronal populations in normal state is mainly in a low frequency band of 0–5 Hz. After Glu-induced seizures, epileptiform activity was concentrated at 0–30 Hz. As the localized epileptiform activity propagates, the distribution and density of power in each frequency band were intensified. A power histogram of electrical activity in normal and epileptiform states is shown in Figure 7b. The power in epileptiform activity is 74.1 ± 5.6 μW, which is four times higher than 18.7 ± 1.3 μW in normal activity with a significant difference (*** *P* < 0.001). Neuronal populations of the epileptogenic zone show an abnormal excitability level. Figure 7c shows the real-time signals simultaneously recorded by the ECoG electrode array. Under normal state, the fluctuation of electrical activity is small, within 0.6 mV. After Glu-induced excitement, the neuronal populations are characterized by spike and wave complex activity and periodic epileptiform activity.

## 4. Discussions

Epilepsy is a neurological disorder with complex pathogenesis [39]. The abnormal electrical signals of cerebral cortex play a crucial role in clinical diagnosis, localization, and treatment of epilepsy. In this work, to study epilepsy, we fabricated a flexible ECoG electrode array for reliable and high spatial resolution recording of cortical neural activities in rats.

A comparison with conventional EEG methods showed that our ECoG electrode array had good flexibility to directly contact with the cerebral cortex, which greatly eliminated the effect of scalp, skull, and dura mater on neural activity recording. We compared the size of electrode sites and the thickness of electrodes with other different µECoG electrodes, which are summarized in Table 1. The smaller size of electrode sites allows for more precise and accurate recordings. In addition, the thinner the electrode, the better it fits the cortex. Orsborn’s team fabricated an array, with 200 µm in electrode diameter and 750–1500 µm in spacing [40]. Kwon’s team used a 4 × 4 transparent Opto-μECoG array for optical neural stimulation on visual cortex, with 200 μm in electrode diameter and 75 μm in electrode thickness [41]. Schendel’s team designed a µECoG device, with 200 μm in electrode diameter, 750 μm in spacing, and 25 μm in electrode thickness [42]. Park’s team applied a graphene-based electrode array on rats, with 150–200 µm in electrode diameter [43]. Our electrode array contained 14 detection sites with a diameter of 60 μm to cover the rat primary somatosensory cortex region. With this distribution, it could locate abnormal neural activity with higher accuracy and finer spatial resolution. Moreover, the thickness of our array was 20 μm. The thin structure had good flexibility to establish reliable contact with the cortex, and the insulation layer was also one of the key factors affecting the neural detection. The thickness of the electrode insulation layer of Chou’s team [25] and Adrega’s team [26] was 5.89 μm and 6 μm, respectively. The insulation layer should be as thin as possible to facilitate close attachment of electrode sites to the nerve cells. The insulation layer of silicon-based implantable microelectrodes is usually a few hundred nanometers (for example 800 nm [30]). The parylene insulation thickness of our electrode array was 1–1.5 μm. Parylene within 1 μm cannot be a dense film. It depends on the quality of the parylene coating machine. Taken together, our electrode array has good performance in terms of spatial resolution and reliable contact, which is essential for obtaining high quality signals. In addition, instead of the invasive electrodes penetrating the cortex, the lightweight structure of our electrode makes it less damaging to the brain tissue. Furthermore, our recording sites are modified by nanoparticles, which enables sensitive sensing interface. The performance of nanoparticles in reducing impedance and improving signal-to-noise ratio (SNR) has been confirmed in another study [44]. Our electrode sites have low impedance and are in close contact with cortical cells, which facilitate high quality detection of cortical signals.

Overexcitation of neuronal populations can cause paroxysmal brain dysfunction, manifested as significant abnormality in electrical signals [45]. In this study, we demonstrated that neuronal populations of primary somatosensory cortex showed hypsarrhythmia rhythm and continuous or periodic spike wave electrical signals at epileptiform state, with an increase in both amplitude and power. We illustrated that GABA had an inhibitory effect on neurons. Other studies have also delivered GABA to treat cortical seizures [46]. In addition, in the frequency domain, delta band showed significant changes, which were highly related to the cortical epileptiform activity. Previous researches based on EEG have reported that delta activity can be an accurate indicator of seizures, and a marker of cortical dysfunction caused by epilepsy brain injury [47,48].

Monitoring changes in amplitude and frequency bands of neural electrical signals could help to detect and locate lesions. The flexible ECoG electrode array provides a promising brain–machine interface platform for further study on epilepsy, seizure detection, and precise localization.

## 5. Conclusions

In this study, we designed and fabricated a flexible ECoG electrode array based on PDMS and parylene for real-time recording of cortical neural activity. The PDMS and parylene substrate showed advantages in flexibility. PtNPs modified microelectrode sites were in close contact with the cerebral cortex, which enabled sensitive sensing interface. Multiple microrecording sites were distributed on the rat primary somatosensory cortex. On the basis of this arrangement, it could locate the abnormal neural activity with higher accuracy and finer spatial resolution. The electrode array could capture cortical electrical activity in real time and reflect changes under drug modulation. Differences in electrical activity between normal and epileptiform states help to detect and locate lesions.

Our flexible ECoG electrode array provides an effective tool for monitoring cortical neural activity. In the future, we plan to further study the electrical characteristics of epileptic activity, which are of great significance for detecting seizures, locating cortical dysfunction regions, and studying the brain network connection.

## Figures and Tables

**Table 1:** Comparison of parameters (material, size, and thickness) in different μECoG electrodes.

Author	Material	Animal	Size	Thickness
Orsborn [40]	Polyimide	non-human primate	200 µm (diameter) 750–1500 µm (spacing)	-
Kwon [41]	Parylene	Rat	200 µm (diameter)	75 μm
Schendel [42]	Parylene	Rat	200 µm (diameter) 750 µm (spacing)	25 μm
Park [43]	Parylene	Rat	150–200 µm (diameter)	-
Ochoa [29]	PDMS and parylene	Rat	90 µm (diameter)	100–200 μm
Chou [25]	PDMS and parylene	-	-	140 μm
Adrega [26]	PDMS	-	200–300 µm (square)	70 μm
Minev [49]	PDMS	Rat	300 µm (diameter)	120 μm
This work	PDMS and parylene	Rat	60 µm (diameter)	20 μm

## Supplementary Materials

The following are available online at https://www.mdpi.com/2072-666X/11/8/732/s1. Figure S1: The power of LFPs in multiple frequency bands (Delta (1–4 Hz), Theta (4–8 Hz), Alpha (8–13 Hz), and Beta (13–30 Hz)) at normal and epileptiform states. The data was from Figure 6. The power from three channels were calculated. The power in delta frequency band, theta frequency band, alpha frequency band and beta frequency band increased from 1.50 μW, 0.62 μW and 0.44 μW, 0.35 μW to 292.47 μW, 116.37 μW, 107.45 μW and 126.86 μW, respectively. Figure S2: The power of LFPs in multiple frequency bands (Delta (1–4 Hz), Theta (4–8 Hz), Alpha (8–13 Hz), and Beta (13–30 Hz)) at normal and epileptiform states. The power from six channels were calculated. The power in delta frequency band, theta frequency band, alpha frequency band and beta frequency band increased from 0.65 μW, 0.50 μW and 0.44 μW, 0.38 μW to 353.27 μW, 101.36 μW, 72.39 μW and 36.98 μW, respectively.
