# Supplementary material for: Flexible Electrocorticography Electrode Array for Epileptiform Electrical Activity Recording under Glutamate and GABA Modulation on the Primary Somatosensory Cortex of Rats
Source: Micromachines (Basel). 2020 Jul 29;11(8):732. doi: 10.3390/mi11080732 (PMC7465452; doi:10.3390/mi11080732)
Supplement: Supplementary file 1 [file micromachines-11-00732-s001.pdf]

micromachines

Supplementary information: Flexible Electrocorticography Electrode Array for Epileptiform Electrical Activity Recording under Glutamate and GABA Modulation on the Primary Somatosensory Cortex of Rats

Xinrong Li [1,2], Yilin Song [1,2], Guihua Xiao [1,2], Jingyu Xie [1,2], Yuchuan Dai [1,2], Yu Xing [1,2], Enhui He [1,2], Yun Wang [1,2], Shengwei Xu [1,2], Lulu Zhang [3], Duli Yu [3], Tiger H. Tao [4] and Xinxia Cai [1,2,*]

1 State Key Laboratory of Transducer Technology, Aerospace Information Research Institute, Chinese Academy of Sciences, Beijing 100190, China; lxr8118@126.com (X.L.); ylsong@mail.ie.ac.cn (Y.S.); xiaoguihua11@126.com (G.X.); xiejingyu16@mails.ucas.ac.cn (J.X.); hongri1991@126.com (Y.D.); xingyu17@mails.ucas.ac.cn (Y.X.); heenhui_iecas@163.com (E.H.); wangyun_iecas@163.com (Y.W.); swxu@mail.ie.ac.cn (S.X.)
2 Department, University of Chinese Academy of Sciences, Beijing 100049, China
3 College of Information Science and Technology, Beijing University of Chemical Technology, Beijing 100029, China; llzhang@mail.buct.edu.cn (L.Z.); dyu@mail.buct.edu.cn (D.Y.)
4 State Key Laboratory of Transducer Technology, Shanghai Institute of Microsystem and Information Technology, Chinese Academy of Sciences, Shanghai 200050, China; tiger@mail.sim.ac.cn (T.H.T.)
* Correspondence: xxcai@mail.ie.ac.cn; Tel.: +86-10-58887193

Received: 22 June 2020; Accepted: 24 July 2020; Published: date

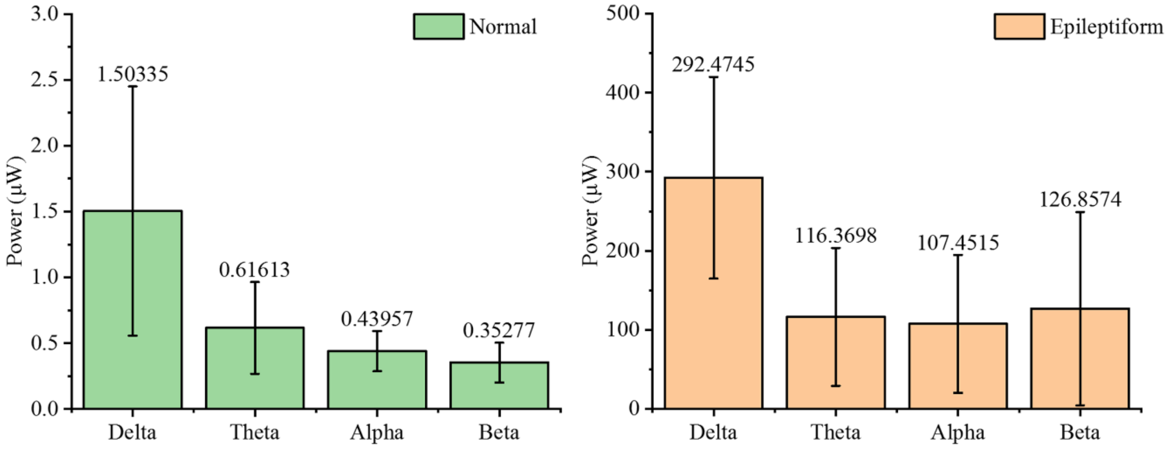

Figure S1. The power of LFPs in multiple frequency bands (delta (1–4 Hz), theta (4–8 Hz), alpha (8–13 Hz), and beta (13–30 Hz)) at normal and epileptiform states. The data was from Figure 6. The power from three channels were calculated. The power in delta frequency band, theta frequency band, alpha frequency band and beta frequency band increased from 1.50 µW, 0.62 µW and 0.44 µW, 0.35 µW to 292.47 µW, 116.37 µW, 107.45 µW and 126.86 µW, respectively.

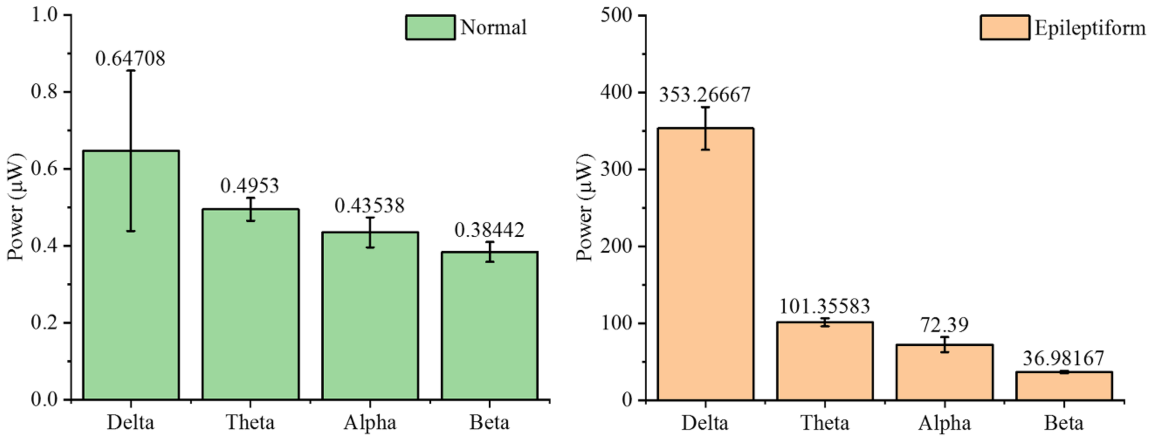

Figure S2. The power of LFPs in multiple frequency bands (delta (1–4 Hz), theta (4–8 Hz), alpha (8–13 Hz), and beta (13–30 Hz)) at normal and epileptiform states. The power from six channels were calculated. The power in delta frequency band, theta frequency band, alpha frequency band and beta frequency band increased from 0.65 μW, 0.50 μW and 0.44 μW, 0.38 μW to 353.27 μW, 101.36 μW, 72.39 μW and 36.98 μW, respectively.
